# Prediction of the degree of pathological differentiation in tongue squamous cell carcinoma based on radiomics analysis of magnetic resonance images

**DOI:** 10.1186/s12903-021-01947-9

**Published:** 2021-11-19

**Authors:** Baoting Yu, Chencui Huang, Jingxu Xu, Shuo Liu, Yuyao Guan, Tong Li, Xuewei Zheng, Jun Ding

**Affiliations:** 1 Department of Radiology, China-Japan Union Hospital of Jilin University, Changchun, 130021 China; 2 Department of Research Collaboration, R&D Center, Beijing Deepwise & League of PHD Technology Co., Ltd, No. 829 of Xinmin Street, Chaoyang District, Beijing, 100080 China

**Keywords:** Tongue squamous cell carcinoma (TSCC), Radiomics, Texture analysis, Degree of pathological differentiation, Magnetic resonance imaging (MRI)

## Abstract

**Background:**

Tongue squamous cell carcinoma (TSCC) is one of the most difficult malignancies to control. It displays particular and aggressive behaviour even at an early stage. The purpose of this paper is to explore the value of radiomics based on magnetic resonance fat-suppressed T2-weighted images in predicting the degree of pathological differentiation of TSCC.

**Methods:**

Retrospective analysis of 127 patients with TSCC who were randomly divided into a primary cohort and a test cohort, including well-differentiated, moderately differentiated and poorly differentiated. The tumour regions were manually labelled in fat-suppressed T2-weighted imaging (FS-T2WI), and PyRadiomics was used to extract radiomics features. The radiomics features were then selected by the least absolute shrinkage and selection operator (LASSO) method. The model was established by the logistic regression classifier using a 5-fold cross-validation method, applied to all data and evaluated using the area under the receiver operating characteristic curve (AUC), accuracy, sensitivity and specificity.

**Results:**

In total, 1132 features were extracted, and seven features were selected for modelling. The AUC in the logistic regression model for well-differentiated TSCC was 0.90 with specificity and precision values of 0.92 and 0.78, respectively, and the sensitivity for poorly differentiated TSCC was 0.74.

**Conclusions:**

The MRI-based radiomics signature could discriminate between well-differentiated, moderately differentiated and poorly differentiated TSCC and might be used as a biomarker for preoperative grading.

## Background

Tongue squamous cell carcinoma (TSCC) is one of the most difficult malignancies to control [1]. It displays particularly aggressive behaviour even at an early stage [2], and despite significant advances in cancer therapeutics over the past 30 years [3], the 5-year survival rate is still unsatisfactory [4]. One reason for this dismal outlook could be the biological propensity for local invasion and the high incidence of cervical lymph node metastasis at the time of diagnosis (40%) [5]. Another reason is that, without consideration of individual differences in genetic and biological behaviour, it potentially results in ineffective treatment in some patients and unnecessary overtreatment in others. Haider et al. [6] and Giraud et al. [7] summarised the application of radiomics and machine learning in head and neck squamous cell carcinomas. They focused on obtaining objective information through non-invasive testing, combined with machine learning to build pathological classification prediction models or conventional prognostic models, guide the selection of treatment, design the scope of surgery, and even guide the comprehensive postoperative treatment. But most studies focused on computed tomography (CT). Our study mainly studied magnetic resonance imaging (MRI). It enriches the application of MRI in machine learning of head and neck squamous cell carcinoma (SCC) cases.

Therefore, it is important to understand the clinical behaviour and outcome of TSCC and focus on individually tailored treatment. Although histological assessment of biopsy or surgical specimens is still the gold standard [8], rapid histological biopsy before surgery may not allow for the evaluation of the characteristics of the entire tumour, which is required for the diagnosis of TSCC. Many studies have concluded that the degree of pathological differentiation is a strong histological feature that correlates with locoregional recurrence [9]. Early comprehensive treatment is needed for patients with poorly differentiated tumours to maximise the therapeutic ratio, avoid unnecessary extended resection and treatment and improve quality of life and prognosis.

Cross-sectional MRI can reveal the extent of locoregional tumour spread, depth of invasion, the extent of lymphadenopathy, and occult metastasis in exquisite anatomical detail [10], so there may be a degree of correlation between the biological heterogeneity of a malignant tumour and the heterogeneity of its image texture. Recent research has shown that some histopathological parameters can be evaluated preoperatively with an MRI [11] or a satisfactory diagnostic biopsy [12]. The degree of pathological differentiation reflects the extent of the malignancy [13]. The poorer the differentiation, the more disordered the tissue and vascular structure and the higher the risk of cancer cells invading the surrounding tissues [14]. Given that biomarkers seen on MRI may reflect the heterogeneity of cancerous tissue, texture analysis of a single biomarker may have increased sensitivity and specificity [15].

Texture analysis is a branch of radiomics based on imaging features and uses a computer algorithm to provide the spatial distribution features of the grey level in an area to quantify the heterogeneity of a tumour [16, 17]. MRI based on imaging radiomics analysis can not only detect and locate the focus but also monitor disease progression and the response to curative treatment. It can also provide information that a biopsy cannot, i.e., the overall heterogeneity of the tumour and the effects of long-term treatment [18, 19]. In recent years, radiomics analysis has played an increasingly bigger role in cancer research, particularly in the identification of imaging biomarkers and clinical management, including classification and staging, evaluation of efficacy, and prediction of the prognosis of brain, breast, prostate, and lung tumours [20–24]. An increasing number of imaging characteristics have been reported to be highly predictive of the degree of pathological differentiation of tumours and to have diagnostic value [25].

In this study, fat-suppressed T2-weighted imaging (FS-T2WI) data and a series of machine learning algorithms were used to build a prediction model, identify imaging markers that could potentially predict the degree of pathological differentiation of TSCC, quantify and visualise the radiomics features extracted from MRI scans, and develop a diagnostic model to guide the selection of individualised diagnostic and treatment methods and improve the outcomes for patients. Therefore, the present study was established to find prognostic or predictive imaging markers that can reflect the pathological differentiation of TSCC and provide more personalised treatment for TSCC to improve the cure rate and reduce the side effects.

## Methods

### Patients

A total of 127 patients with TSCC were randomly divided into a primary cohort (72 patients: 23 well-differentiated, 28 moderately differentiated and 21 poorly differentiated) and a test cohort (55 patients: 22 well-differentiated, 20 moderately differentiated and 13 poorly differentiated). The cohorts included 92 men and 35 women, with a mean age of 58.6 (range 38–75) years, with biopsy-proven TSCC for which preoperative MRI scans were acquired between June 2016 and October 2020. A retrospective analysis was performed to determine the degree of pathological differentiation. The clinical characteristics of the primary cohort and the test cohort are shown in Table 1. The inclusion criteria were to have a case of biopsy-proven TSCC with complete clinical data and absence of concomitant disease, and the ability to cooperate with an MRI examination. The exclusion criteria were as follows: no definitive postoperative information on pathological characteristics, local or systemic treatment before surgery, a minimum tumour diameter < 5 mm (not amenable to placement of a region of interest [ROI]), and poor MRI quality for post-processing due to artefact.

**Table 1:** Patient characteristics in the primary and test cohort (n = 127)

	Pathological differentiation degree of TSCC							
	Primary cohort				Test cohort			
	Poorly differentiated (n = 23)	Moderately differentiated (n = 28)	Well differentiated (n = 21)	P-value	Poorly differentiated (n = 13)	Moderately differentiated (n = 20)	Well differentiated (n = 22)	P-value
Sex								
Male	21	24	17	0.610	9	9	12	0.674
Female	2	4	4		4	11	10	
Age, years								
Range	39–75	43–75	38–75	0.514	47–68	53-72	34-62	0.694
Average	56.8	59.0	59.8		55.4	61.2	49.8	
Localization of tumor								
Tip	9	8	1	0.094	3	6	8	0.059
Body	9	10	12		7	6	6	
Root	5	10	8		3	8	8	
Pain								
Yes	18	20	20	0.691	10	13	13	0.725
No	5	8	1		3	7	9	
Margin								
Well-circumscribed	8	8	3	0.094	3	8	18	0.053
Ill-defined	15	20	18		10	12	4	
Cystic degeneration								
Yes	14	13	14	0.330	7	12	5	0.191
No	9	15	7		6	8	17	
Sublingual gland duct dilatation								
Yes	10	12	6	0.514	3	7	2	0.483
No	13	16	15		10	13	20	
Lymphatic metastasis								
Yes	12	6	4	0.021	10	5	3	0.000
No	11	22	17		3	15	19	
HPV								
Positive	14	22	15	0.383	9	11	15	0.601
Negative	9	6	6		4	9	7	

Notes: A P-value < 0.05 was considered to indicate a statistically significant difference. *age’s test^ independent-samples t-test, others’s test^chi-square test

### MRI and texture analysis

**Fig. 1:**
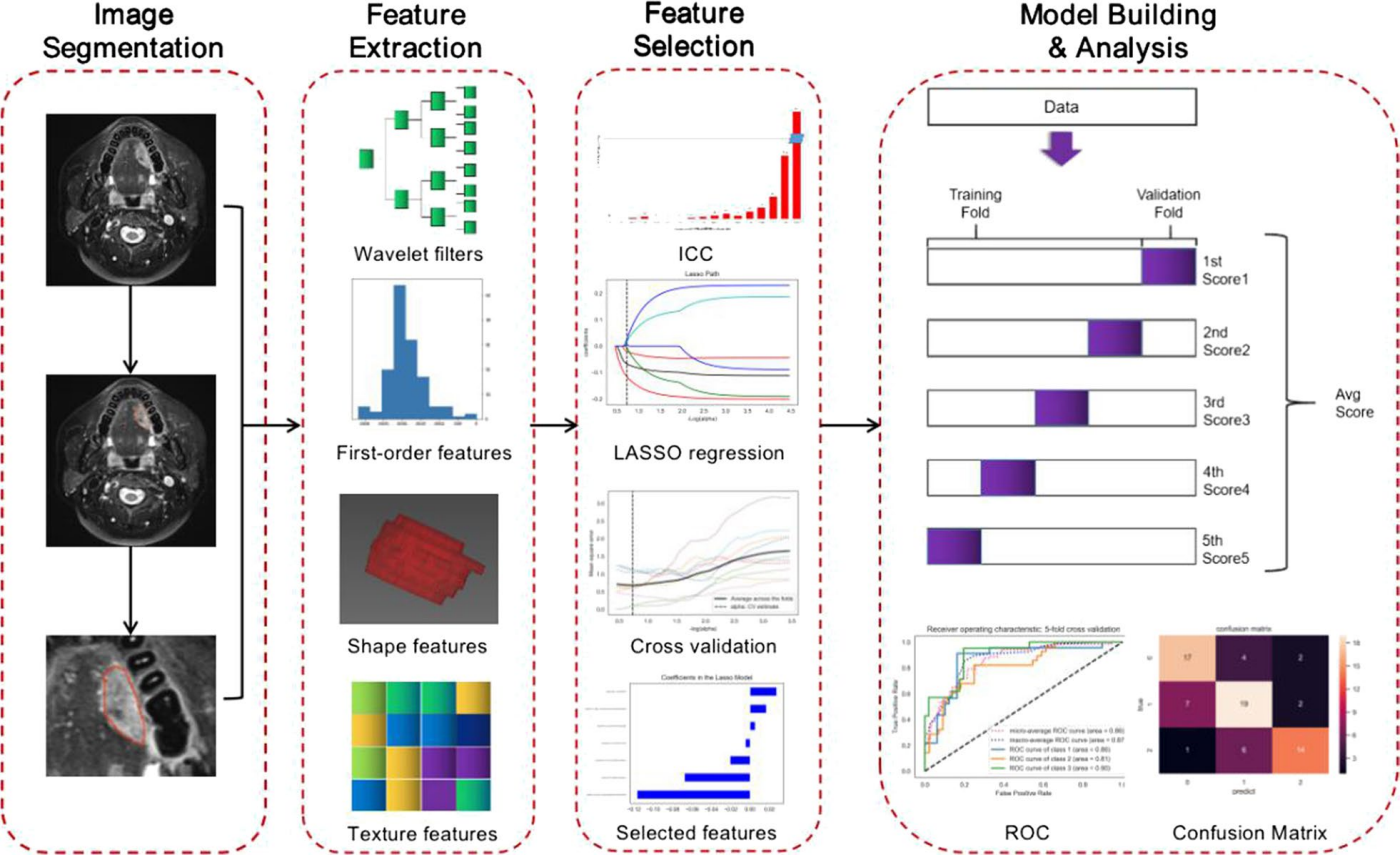
Flow chart of radiomics analysis. From left to right, manual segmentation to obtain voxel-based region of interest in a three-dimensional slice, extraction of radiomics features using Pyradiomics software, selection of features using LASSO regression, development of the model, and evaluation of diagnostic performance using ROC analysis

All the MRI examinations were performed using a 1.5-T Avanto scanner (Siemens Healthineers, Erlangen, Germany) with an 8-channel phased-array neck coil. The patient’s head was secured using a relaxing cushion, ensuring that the shoulders were in contact with the lower part of the coil. Non-contrast axial FS-T2WI sequence acquired in multiple breath-holds were obtained using the following parameters: a repetition/echo time of 5080/87 ms, a slice thickness/interslice gap of 4.0/0.4 mm, 20 slices, and a matrix of 256 × 256. This study used the Dr Wise Multimodal Research Platform (https://keyan.deepwise.com) (Beijing Deepwise & League of PHD Technology Co., Ltd, Beijing, China) for texture analysis, including image annotation. For the extraction of features, an open-source python package called PyRadiomics (2.1.0), a platform that supports feature extraction in both two and three dimensions and can be used to calculate single values per feature for an ROI (‘segment-based’) or generate feature maps (‘voxel-based’), was used. The steps in the texture analysis are shown in Fig. 1.

### Delineation of tumour ROI

All scans were retrospectively reviewed, loaded, and processed in the original DICOM format and then transmitted to the post-processing workstation. The tumour regions in the primary dataset were labelled manually by two experts. In cases of discordance, the opinion of a third radiologist was requested, and a consensus was reached through discussion. Finally, the volume of interest was obtained for feature extraction and quantification. An axial FS-T2WI scan was selected as the labelling image. It was possible to classify the tumour tissue based on the principle of not exceeding the tumour boundary.

### Extracting features from MRI scans

The high-pass or low-pass wavelet filter and Laplacian of Gaussian filter with different λ parameters were used to pre-process the original images. Each stage of wavelet filtering generated eight decompositions, and all possible combinations of high-pass or low-pass filters (wavelet_LLH, wavelet_LHL, wavelet_LHH, wavelet_HLL, wavelet_HLH, wavelet_HHL, wavelet_HHH, and wavelet_LLL) and four Laplacian of Gaussian filters (λ = 2, 3, 4, 5) were applied in all three dimensions for pre-processing. The features extracted from the original images included their first-order features based on the pixel value, the shape features of the tumour, and the internal and surface texture features extracted from each ROI, including the grey level co-occurrence matrix (GLCM), grey level run length matrix (GLRLM), grey level size zone matrix (GLSZM), and grey level dependence matrix (GLDM). In total, 1132 radiomics features were extracted from each ROI, and a standardised Z-score was then obtained by subtracting the average value and dividing by the standard deviation. Features showing poor consistency between different groups were removed by calculating the intraclass correlation coefficient (ICC). Features with an ICC > 0.75 were selected and modelled. Finally, the least absolute shrinkage and selection operator (LASSO) algorithm was used for feature reduction and selection. The most important feature with a coefficient that was not zero was identified for modelling and improving the model’s performance. The LASSO method uses a shrinking (regularisation) process whereby it penalises the coefficients of the regression variables, shrinking some of them to zero. Variables that still have a non-zero coefficient after the shrinking process are selected to be part of the model. The goal of this process was to minimise prediction error.

### Establishment of the model

The logistic regression (LR) classifier was used to establish the diagnostic model by the 5-fold cross-validation method. The establishing of diagnostic model was performed by primary cohorts, thereafter the created model was tested in test cohorts. After all training and testing, the performance of the model was evaluated by the average value of five tests. In this study, the generalisation properties of the learning algorithm were focused on for multiclass classification problems, and the confusion matrix of each classifier was used as a measure of its quality. A confusion matrix is useful for evaluating the ability of classifiers to classify multiclass objects in addition to receiver operating characteristic (ROC) curves. The macro-average and micro-average are indexes to measure the classifier. Macro-average indicators are more affected by small categories than micro-average indicators. Therefore, the AUC comparison in this experiment mainly focuses on the micro-average. The accuracy score—the ratio of the correctly classified samples to all samples—was also used to evaluate the predictive performance of the model. Finally, a model was computed by using selected features.

### Statistical analysis

The classification model was built using the Scikit-learn software package (version 0.20.3). Matplotlib (version 3.1.0) was used to draw the ROC curves. The statistical analysis of general data was performed using SPSS for Windows version 16.0 (IBM Corp., Armonk, NY, USA). Chi-square tests were used to detect differences in the categorical variables between groups. Group differences in quantitative variables were examined using independent-samples *t*-tests. *P  *< 0.05 was considered statistically significant. Comparing AUCs of Machine Learning Models was done by a DeLong’s test, testing whether one machine learning model’s test set performance differs significantly from the test set performance of an alternative model.

## Results

### Patients and radiomics features

**Fig. 2:**
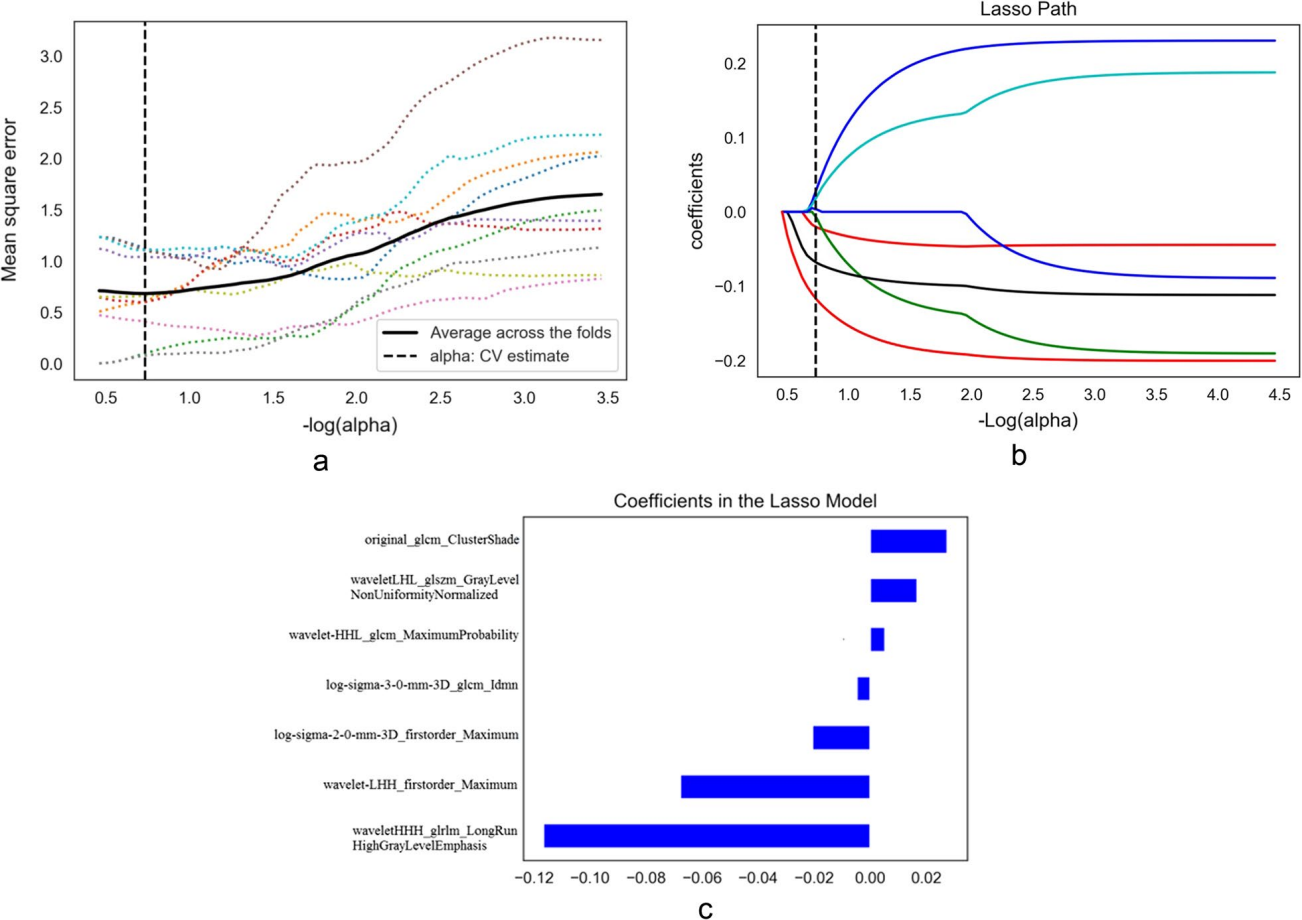
**a** The MSE path in the LASSO algorithm. The dotted lines in different colors indicate each group of cross-validation samples corresponding to different -log(alpha) with a different MSE. The black solid line is the mean value of five MSE groups. The best alpha is the value with the lowest MSE. Notes: The optimization objectives of LASSO are as follows: CV, LASSO, least absolute shrinkage and selection operator; MSE, mean square error. **b** LASSO coefficient solution path for the seven features. **c** Coefficients in the LASSO model of the seven features. The vertical axis represents the seven key features for modeling. The transverse axis represents the relative weight of these features

**Fig. 3:**
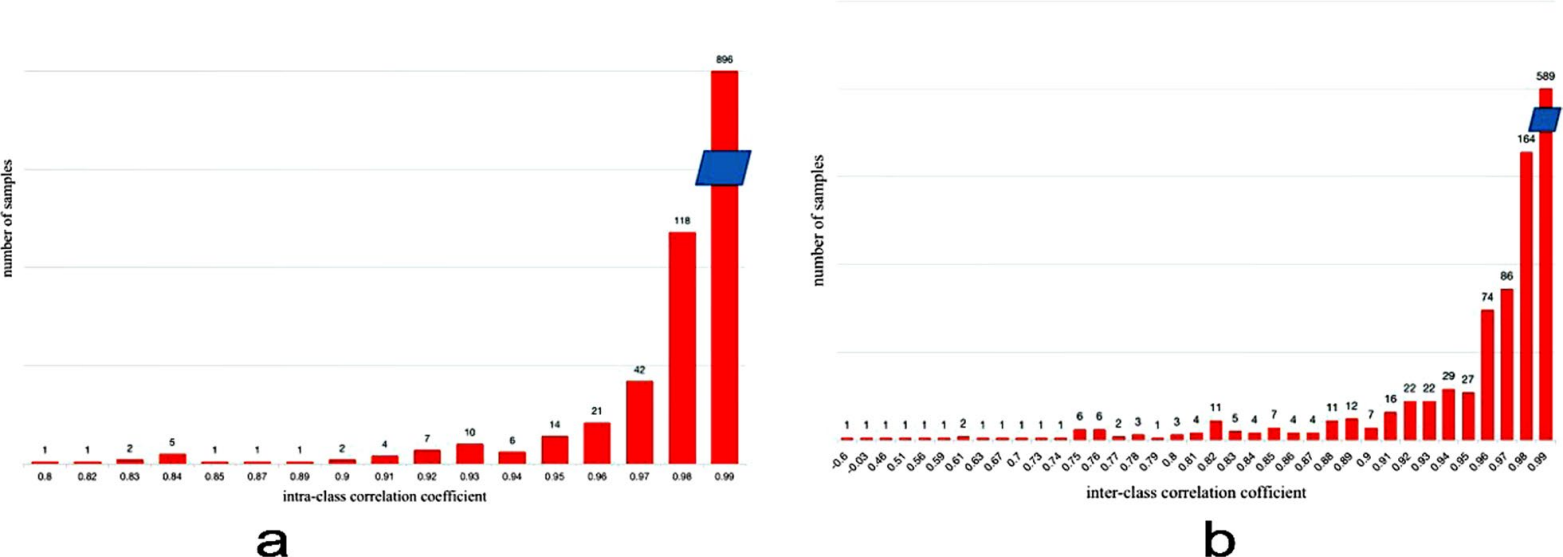
Intraobserver (**a**) and interobserver (**b**) consistency (ICC)

The characteristics of the 127 patients with TSCC are summarised in Table 1. There were significant differences in the rates of lymphatic metastasis (*P* < 0.05), based on the degree of pathological differentiation of TSCC in the cohort. There was no significant difference in sex, age, localisation of the tumour, pain, margin, cystic degeneration, sublingual gland duct dilatation or human papillomavirus (HPV), based on the degree of pathological differentiation. A total of 1132 imaging features, including 234 first-order features, 14 shape features and 884 texture features, were extracted from the original images through wavelet and Laplacian of Gaussian filters and selected by the LASSO algorithm. The key relevant features were selected by the 5-fold cross-validation method, as shown in the mean square error (MSE) path of the LASSO algorithm (Fig. 2a). The best alpha value was 0.18484, and the -log(alpha) value was 0.73320. The vertical axis of the path graph of the coefficient solution (Fig. 2b) represents the coefficients of each feature in the LASSO model, which change with the change in alpha value. Based on the best alpha value, the coefficients corresponding to the different features were identified. Features with a coefficient that was not 0 were selected, and finally, seven of the 1132 features were selected for modelling. The feature list and coefficient chart showed two first-order features (wavelet-LHH_first-order_maximum and log-sigma-2-0-mm-3D_first-order_maximum) and five texture features (wavelet-HHH_glrlm_LongRunHighGreyLevelEmphasis,log-sigma-3-0-mm-3D_glcm_Idmn,wavelet-HHL_glcm_MaximumProbability, wavelet-LHL_glszm_GreyLevelNonUniformityNormalised, and original_glcm_ClusterShade) (Fig. 2c). The intraobserver and interobserver consistency of the annotation images were good (Fig. 3). The consistency of all features of the selected model is greater than 0.75.

### Establishment and evaluation of the model

#### 1) Performance evaluation of the prediction model in the primary cohort

**Fig. 4:**
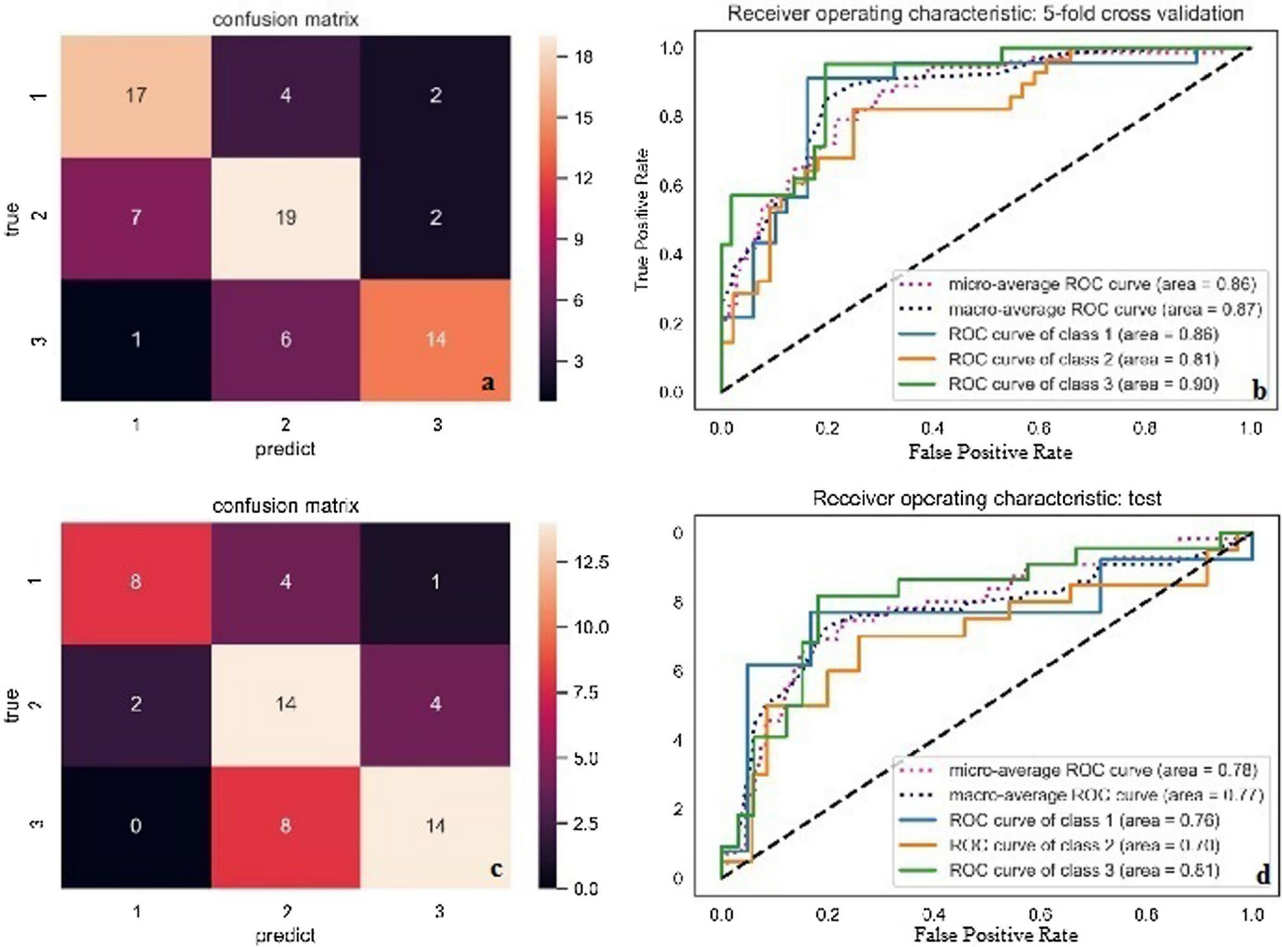
Performance assessment of prediction model in primary set and testset. Confusion matrix ROC curve analysis in primary cohort (**a** and **b**) and test cohort (**c** and **d**). The solid lines in different colors indicate that the ROC curve for each class of TSCC correspond to a different AUC, which represents the positive rate of prediction of the degree of pathological differentiation. The dotted lines in different colors indicate the ROC curves of the micro-average and macro-average. Notes: Numbers 1, 2, and 3, represent poorly, moderately, and well differentiated TSCC, respectively

The prediction model’s performance was first assessed in the testing sets with the equilibrium of the class distribution and balanced data. The results are shown in Table 2. In terms of the accuracy of the classification, the confusion matrix results confirmed that there was consistency between the predicted and actual results, suggesting a better performance of the model in the classification of multiclass objects (Fig. 4a). ROC curve analysis also verified that the model could predict and distinguish the degree of pathological differentiation of TSCC with high accuracy of 0.81–0.90 (all AUC > 0.80). The diagnostic effect of this prediction model on differentiation was more than 80%. The micro-average AUC of the LR model in the 5-fold cross-validation of the primary cohort was 0.86, tending towards the upper left corner and far from the diagonal (Fig. 4b). Moreover, in the LR model, the AUC for well-differentiated TSCC was 0.90, suggesting that this model had the highest accuracy for predicting and distinguishing well-differentiated TSCC.

**Table 2:** The results of primary set and test set

	Precision	Sensitivity	Specificity	F1-score	Support
Primary set					
Poorly	0.68	0.74	0.84	0.71	23
Moderately	0.66	0.68	0.77	0.67	28
Well	0.78	0.67	0.92	0.72	21
Macro avg	0.70	0.69	0.84	0.70	72
Weighted avg	0.70	0.69	0.84	0.69	72
Test set					
Poorly	0.80	0.61	0.95	0.69	13
Moderately	0.54	0.70	0.78	0.61	20
Well	0.74	0.66	0.85	0.68	22
Macro avg	0.69	0.65	0.82	0.66	55
Weighted avg	0.68	0.65	0.80	0.66	55

Notes: F1-score = 2PR/(P+R) (P, precision; R, recall)

#### 2) Performance evaluation of prediction model in a test cohort

In addition to the internal testing above, external validation for the performance of the prediction model was also performed by using a test cohort without pre-processing or the equilibrium of the class distribution. The machine learning algorithms in the test cohort were the same as those used in the primary cohort. In terms of classifier performance in the test cohort, the confusion matrix and ROC curves both indicated a high classification accuracy of the model, with an AUC value of 0.70–0.81. ROC curves also revealed that the model could distinguish the degree of pathological differentiation of TSCC with high accuracy (Fig. 4c, d). Ultimately, the prediction model that used seven optimal characteristics was validated to be significantly effective in predicting the degree of pathological differentiation of TSCC.

## Discussion

In this study, the texture features of 127 patients with biopsy-proven TSCC were extracted and analysed. Correlations were sought by comparing these features with histopathological features that were determined postoperatively. Tumour characteristics that suggest the degree of pathological differentiation are difficult to distinguish accurately by visual observation alone. We found that compared with DWI sequence images, the correlation between T2WI imaging features and pathological differentiation of TSCC was more obvious in patients with TSCC. The prediction results of the T2WI model and DWI model were analysed by DeLong’s test. In the training and the validation set, we use DeLong’s test to demonstrate that the T2WI model has a significantly different AUC from the DWI model with *P* < 0.05. In general, classification models with AUC values of 1.00–0.90 and 0.90–0.80 are regarded as excellent and good, respectively [26]. The micro-average AUC values for the prediction models constructed by the LR in the 5-fold cross-validation of the primary cohort reached 0.86, and the AUC and specificity values for well-differentiated tumours was better (> 0.90), indicating that the diagnosis model can accurately distinguish well-differentiated TSCC. This model also had the highest sensitivity for the diagnosis of poorly differentiated tumours. In terms of the test cohort, classifier performance indicated a high classification accuracy of the model. Parmar et al. [27] compared different classifiers to predict overall survival based on a set of 440 radiomic features extracted from 231 head and neck cancer (HNSCC) primary tumour lesions in contrast-enhanced CT images. In this study, we also compared different classifiers. The primary and test cohort indicated a high classification accuracy of the model. Therefore, the radiomics analysis described here has a strong ability to predict the degree of pathological differentiation of TSCC.

The most recent World Health Organization guidelines [28] recommend using cell differentiation to grade head and neck carcinomas. Lower tumour heterogeneity is likely associated with a lower histological grade [29]. In the present study, most cases were classified as moderately differentiated, consistent with what other studies have observed for oral squamous cell carcinoma (OSCC) [30]. Moreover, similar to results reported by Jing et al. [31], poorly differentiated tumours showed a statistically significant relationship with recurrence (*P* = 0.043). Therefore, poor differentiation is a known risk factor for treatment failure in patients with tongue cancer [32]. It is important to determine the degree of pathological differentiation preoperatively to assess the prognosis. Fujima et al. [33] studied the utility of the MRI histogram and texture analysis in head and neck malignancies; however, it is limited to the correlation analysis between first-order features and DWI values, and no external verification was carried out. Compared with the study of Ren et al., the T2WI sequence is the research object in this study. It not only reflects the internal characteristics of lesions more comprehensively but also the key sequence of disease differentiation. The results of this study show that T2WI has greater advantages in distinguishing different degrees of differentiation of TSCC. For TSCC, larger resections generally result in a worse functional outcome [34].

Prognostic and predictive markers hold the promise of allowing more personalised treatment of TSCC to improve cure rates and minimise side effects. In patients followed closely for N_0_phase, 20–30% will subsequently develop cervical lymph node metastases. A 40% incidence of micrometastatic disease was still found when elective neck dissection was performed in patients with T1 and T2 TSCC [35]. Most of the patients were poorly differentiated. HPV positivity is a strong independent prognostic factor for favourable outcomes and overall survival (OS) in patients with oropharyngeal SCC (OSCC) [36, 37]. Since 2015, multiple studies have demonstrated the association of radiomic features with HPV status in HNSCC. While Buch et al. [38] and Fujita et al. [39] examined the association of individual texture features with HPV status, other groups have designed machine learning classification models for HPV prediction in HNSCC. HPV is positive in most TSCC, but there was no significant difference in HPV status based on the degree of pathological differentiation. Therefore, these present findings have the potential to impact the clinical management of early TSCC. Excessive staging may lead to the loss of opportunity for effective surgical treatment, and too low clinical staging may result in ineffective or even harmful therapy. However, an accurate judgement of the degree of pathological differentiation before surgery may improve this situation. The performance of radiomics analysis varies depending on the MRI scanner, imaging parameters and tumour delineation method used [40, 41]. MRI scans acquired by one type of scanner and imaging parameters from the entire dataset were used to reduce the influence of these variations on performance [35]. However, in terms of delineation, the consistency within and between groups was analysed using the ICC to avoid interobserver and intraobserver differences. The results of independent verification suggested that reproducible radiomic features for observing delineation variability should be investigated to obtain high prediction performance when using different data. In this study, rather than measuring the largest diameter of the tumour slice, the whole tumour volume was measured. This can extract tumour features more efficiently, thereby offering an opportunity to overcome the limitations of visual image interpretation and refine the characteristics of different tumour regions [42].

In this study, an intensive search for non-invasive imaging biomarkers was undertaken, and a prediction model that can not only detect imaging information hidden in focus but can also distinguish the degree of differentiation of that focus, which has high clinical value, was used. At present, the research on texture analysis of head and neck tumours is relatively limited. Texture analysis and prediction of the differentiation degree of TSCC by MRI are not only a breakthrough and innovation in the diagnosis of TSCC but also of significance in clinical diagnosis and treatment. The advantages of MRI over CT scans as a non-invasive predictive tool have been confirmed in many research studies [43]. The MRI has high soft tissue contrast and can reflect the internal information of the lesion to a greater extent. This study also had some limitations. The study data was limited and obtained from a single centre, the ROI still depends on a semiquantitative feature extraction method, the edge and contour of the tumour are affected by the experience of the evaluator, and the diagnosis model still needs to be used in conjunction with other important diagnostic indicators, with the cases increasing.

## Conclusions

In conclusion, in this study, we constructed a radiomics model based on MRI that can noninvasively predict and differentiate the degree of pathological differentiation of TSCC, especially highly differentiated TSCC. This radiomics model can be used for precision medicine and improve clinical treatment strategies. High-throughput radiomics data are extracted to establish a model to predict the differentiation degree of TSCC, which may be a method to evaluate imaging biomarkers in patients with TSCC.

## Data Availability

All data generated or analyzed during this study are included in this published article.
